# Burden of headache disorders in China, 1990–2017: findings from the Global Burden of Disease Study 2017

**DOI:** 10.1186/s10194-019-1048-2

**Published:** 2019-11-07

**Authors:** Chengye Yao, Yu Wang, Lijun Wang, Yunning Liu, Jiangmei Liu, Jinlei Qi, Yun Lin, Peng Yin, Maigeng Zhou

**Affiliations:** 1 Department of Neurology, Union Hospital, Tongji Medical College, Huazhong University of Science and Technology, Wuhan, 430022 China; 2 Department of Anesthesiology, Institute of Anesthesia and Critical Care Medicine, Union Hospital, Tongji Medical College, Huazhong University of Science and Technology, Wuhan, 430022 China; 3 National Center for Chronic and Noncommunicable Disease Control and Prevention, Chinese Center for Disease Control and Prevention, 27# Nanwei Rd, Xicheng District, Beijing, 100050 China

**Keywords:** Headache disorders, Migraine, Tension-type headache, Prevalence, Years lived with disability, China, Global Burden of Disease Study

## Abstract

**Background:**

Headache has emerged as a global public health concern. However, little is known about the burden from headache disorders in China. The aim of this work was to quantify the spatial patterns and temporal trends of burden from headache disorders in China.

**Methods:**

Following the general analytic strategy used in the 2017 Global Burden of Disease study, we analyzed the prevalence and years lived with disability (YLDs) of headache and its main subcategories, including migraine and tension-type headache (TTH), by age, sex, year and 33 province-level administrative units in China from 1990 to 2017.

**Results:**

Almost 112.4 million individuals were estimated to have headache disorders in 1990 in China, which rose to 482.7 million in 2017. The all-age YLDs increased by 36.2% from 1990 to 2017. Migraine caused 5.5 million YLDs, much higher than TTH (1.1 million) in 2017. The age-standardized prevalence and YLDs rate of headache remained stable and high in 2017 compared with 1990, respectively. The proportion of total headache YLDs in all diseases increased from 1990 to 2017 by 5.4%. A female preponderance was observed for YLDs and the YLDs were mainly in people aged 20~54 years.

**Conclusions:**

Headache remains a huge health burden in China from 1990 to 2017, with prevalence and YLDs rates higher in eastern provinces than western provinces. The substantial increase in headache cases and YLDs represents an ongoing challenge in Chinese population. Our results can help shape and inform headache research and public policy throughout China, especially for females and middle-aged people.

## Introduction

Headache disorders are almost the most prevalent, burdensome and costly diseases in the world. From a public-health perspective, the headache disorders, mostly migraine and tension-type headache (TTH), have emerged as major global public health concerns, lead to widespread health loss, impaired quality of life and much loss of productivity [1–3]. Migraine was ranked as the second cause for years of life lived with disability (YLDs) in 2016 in the world [2]. In China, headache is also a major health problem, greatly affecting quality of life. With a population of over 1.3 billion, 20% of the total in the planet, it is estimated that China may have the largest headache population worldwide. However, previous epidemiological studies for headache burden were of small sample size, limited localities, high rates of under-diagnosis and misdiagnosis [4, 5]. Furthermore, the burden of headache is easily to be wholly ignored, partly because this disorder is nonfatal. However, headache disorders have impact on a large amount of issues like work or school activities and family life, and may normally be related to long-lasting or objective disability, especially for migraine [6]. The fact that most people experienced headaches from time to time may have hindered the realization that headache disorders are great burden for the quality of life for people who suffered from the condition. Besides, the burden of headache disorders, is multifaceted and fragmented. A comprehensive study on burdens caused by headache disorders is therefore meaningful for populous countries such as China to understand the magnitude of burden and inform the current national or subnational responses that can support the health care system in public-health policies and provide guidance for disease-preventative and health-promoting strategies in future. The Global Burden of Diseases, Injuries, and Risk Factors (GBD) study is such an effort of a worldwide collaboration aimed at quantifying various health metrics of loss to diseases and injuries. The GBD data can produce consistent, transparent, and up-to-date estimates of disease incidence, prevalence, mortality, and other metrics of the disease burden at macro-level (ie, global and national) and meso-level (ie, subnational) geographic scales [7].

Thus, our study aims to describe the current status and the spatial patterns and temporal trends of burden caused by headache from 1990 to 2017 in China, based on the results of the GBD 2017 study.

## Methods

All analyses were based on China data in the GBD study 2017. Details on the data, approaches to enhancing data quality and comparability, and statistical modelling and metrics for the GBD 2017 were published elsewhere [8, 9]. In the main text of this article, a brief overview specific for the health burden estimation of headache disorders, migraine and TTH is presented.

### Definitions

We analyzed data from 33 province-level administrative units in China, including 31 mainland provinces, municipalities, autonomous regions, and the Hong Kong and Macao Special Administrative Regions (SAR), all of which were referred to as provinces in the remainder of this study.

In GBD, to fully analyze the effect of disease burden on a population, disability-adjusted life years (DALYs) were used as a standardized metric to measure morbidity and mortality, which are the sum of years of life lost (YLLs) to premature mortality and YLDs. DALYs for headaches are equivalent to YLDs because GBD does not estimate any deaths from headache disorders as the underlying cause. YLDs for each headache disorder are calculated from its prevalence and corresponding metrics, so prevalence is an important metric for disease burden of headache disorders.

In the GBD 2017 cause hierarchy, headache disorders are on Level 3, under neurological disorders (Level 2) and non-communicable diseases (Level 1). Headache disorders are subdivided to migraine and TTH (Level 4), and no further subdivision exists under Level 4. In GBD 2013 and GBD 2015, medication overuse headache was treated as an individual disorders [2], but in GBD 2017 it was considered a secondary headache [8], which occurs almost exclusively in patients with either migraine or TTH [10]. Therefore, the burden of medication overuse headache was added to the burden estimations for the headache disorders on Level 4.

### Prevalence and YLDs estimates

The detailed descriptions of the modeling strategy for point prevalence and YLDs estimation and validation in GBD 2017 have been described previously [8]. Prevalence data were matched by headache type, age, sex, year and location, from the data sources including published population-based studies of prevalence and survey data [2, 8]. We only included the studies with headache diagnosis based on the International Classification of Headache Diseases-3 (ICHD-3) beta criteria [11]. The prevalence reflects the individuals in the population who have had at least one episode in the past 12 months fulfilling ICHD-3 beta criteria. Disease Modeling-Meta regression 2.1 (DisMod-MR 2.1), was developed to address the challenges in estimating the point prevalence and YLDs outcomes in all regions and countries, and estimates were obtained in this way also for regions where no relevant headache studies had been done. YLDs are estimated as the product of prevalence and the mean time patients spend with that type of headache (or sequelae) multiplied by their corresponding disability weights which quantify the relative severity of sequelae as a number between 0 (representing full health) and 1 (representing death) [2, 12]. Further details on the methods of prevalence and YLDs estimates were provided in the Additional file 1: Method.

Estimates were made for both sexes and 16 age groups ranging from 0 to 5 years of life to older than 80 years in China and 33 provinces from 1990 to 2017. To interpret the changes of YLDs and prevalence in a broader context, the percentage changes from 1990 to 2017 were analyzed and presented. We then assessed the age-standardized estimates and compared the estimates with global average level. The uncertainty interval (UI) was calculated from the standard errors generated from the input data. All estimates were produced with 95% UIs.

## Results

As shown in Table 1, we estimated that 482,691,000 cases with headache disorders in China in 2017, accounting for 34.7% of the total Chinese population. The YLDs were 6609,000 person years from headache disorders in China in 2017. The percentage change for YLDs and prevalence was 36.2% (95% UI: 31.8%, 41.5%) and 30.3% (95% UI: 24.8%, 36.2%) respectively. As for migraine and TTH, the cases and YLDs increased significantly from 1990 to 2017 by over 30%. A female preponderance was also observed for the burden from overall headache disorders, as well as migraine and TTH.

**Table 1:** All-age YLDs and prevalence for headache disorders and their percentage change by sex in China,1990–2017

Subcategories	All-Age YLDs, No. in Thousands (95% UI)			All-Age Prevalence, No. in Thousands (95% UI)		
	1990	2017	Change (%)	1990	2017	Change (%)
Headache disorders						
Total	4853 (3119 - 6992)	6609 (4275 - 9560)	36.2%(31.8%, 41.5%)	112,362 (103,589 - 121,775)	482,691 (447,400 - 521,213)	30.3%(24.8%, 36.2%)
Male	1842 (1195 - 2717)	2456 (1601 - 3634)	33.4%(28.6%, 39.0%)	164,930 (149,995 - 181,254)	211,593 (194,386 - 231,838)	28.3%(21.9%, 35.1%)
Female	3011 (1938 - 4298)	4153 (2711 - 5953)	37.9%(32.7%, 44.4%)	205,379 (190,393 - 222,236)	271,098 (252,422 - 290,621)	32.0%(26.3%, 38.5%)
Migraine						
Total	4021 (2487 - 6003)	5476 (3431 - 8159)	36.2%(31.3%, 42.1%)	370,309 (340,750 - 402,078)	151,600 (139,966 - 163,357)	34.9%(30.1%, 40.4%)
Male	1456 (891–2261)	1937 (1187 - 2958)	33.0%(27.4%, 39.2%)	39,185 (36,065 - 42,498)	51,413 (47,465 - 55,703)	31.2%(25.6%, 37.2%)
Female	2564 (1601 - 3791)	3539 (2233 - 5206)	38.0%(32.1%, 45.1%)	73,176 (67,155 - 79,366)	100,186 (92,352 - 108,224)	36.9%(30.9%, 43.4%)
TTH						
Total	832 (463–1338)	1133 (643–1807)	36.2%(28.8%, 43.9%)	300,356 (267563–337,449)	388,965 (347,287 - 435,249)	29.5%(21.8%, 38.3%)
Male	385 (213–621)	519 (291–823)	34.7%(27.1%, 43.9%)	139,018 (122896–157,164)	177,799 (158,236 - 200,234)	27.9%(19.6%, 36.9%)
Female	447 (250–716)	614 (349–979)	37.4%(29.5%, 46.0%)	161,338 (143591–181,532)	211,166 (189,154 - 235,260)	30.9%(22.7%, 40.8%)

Note: *YLDs* years lived with disability; data shown as rate (95% uncertainty interval)

The age-standardized prevalence rate and YLDs rate for headache, percentage and ranking of YLDs in YLDs from all causes globally and for China from 1990 to 2017 are displayed in Table 2. In China, the age-standardized prevalence and YLDs rate (per 100,000) for headache disorders was 30,936 (95% UI: 28,627 - 33,431) and 401 (95% UI: 259–577) in 2017, remaining stable compared with 1990. As for migraine and TTH, in 2017, the age-standardized YLDs rate (per 100,000) from migraine with 331 (95% UI: 207–495) was higher than the YLDs rate for TTH with 70 (95% UI: 39–113). The age-standardized prevalence and YLDs rate in China was lower than the global average. The YLDs from headache accounted for 6.3% worldwide and 4.4% in China among total YLDs from all causes in 2017. Headache disorders still ranked as the second leading cause of YLDs at the global level in both 1990 and 2017, which was higher than the headache disorder ranking in China (ranked fourth in 1990 and eighth in 2017).

**Table 2:** The age-standardized prevalence and YLDs rate for headache in China and globally

Variable	World			China		
	1990	2017	^b^change(%)	1990	2017	^b^change(%)
^a^Prevalence rate						
Headache disorders	39,071 (36,888-41,587)	39,192 (37,025-41,599)	0.3%(− 0.2%,0.9%)	30,860 (28,665-33,381)	30,936 (28,627-33,431)	0.2%(−1.9%,2.4%)
Migraine	16,543 (15,363-17,829)	16,828 (15,638-18,119)	1.7%(1.1%,2.4%)	9267 (8602-10,008)	9211 (8527-9921)	−0.6%(−3.2%,2.0%)
TTH	29,917 (27,116-33,139)	29,810 (27,057-32,943)	−0.4%(−1.1%,0.5%)	25,099 (22,595-28,046)	25,233 (22,611-28,148)	0.5%(−2.3%,3.5%)
^a^YLDs rate						
Headache disorders	677 (442–953)	687 (449–971)	1.4%(0.8%,2.1%)	402 (259–581)	401 (259–577)	−0.2%(− 2.5%,2.2%)
Migraine	587 (370–850)	597 (378–866)	1.7%(1.1%,2.4%)	332 (206–495)	331 (207–495)	−0.3%(−2.8%,2.4%)
TTH	90 (52–143)	90 (51–142)	−0.7%(−1.5%,0.1%)	70 (40–112)	70 (39–113)	0.0%(−2.4%,2.4%)
Proportion of YLDs						
Headache disorders	6.0%(4.4%,7.8%)	6.3%(4.7%,8.2%)	5.5%(4.4%,6.5%)	4.2%(3.1%,5.5%)	4.4%(3.3%,5.8%)	5.4%(2.7%,8.0%)
^c^Ranking of YLDs						
Headache disorders	2	2		4	8	

Note: *YLDs* years lived with disability

^a^Age-Standardized rate (1/100,000) and data shown as rate (95% UI); ^b^ percentage change of the metrics from 1990 to 2017; ^c^ranking of YLDs in all YLDs

**Fig. 1:**
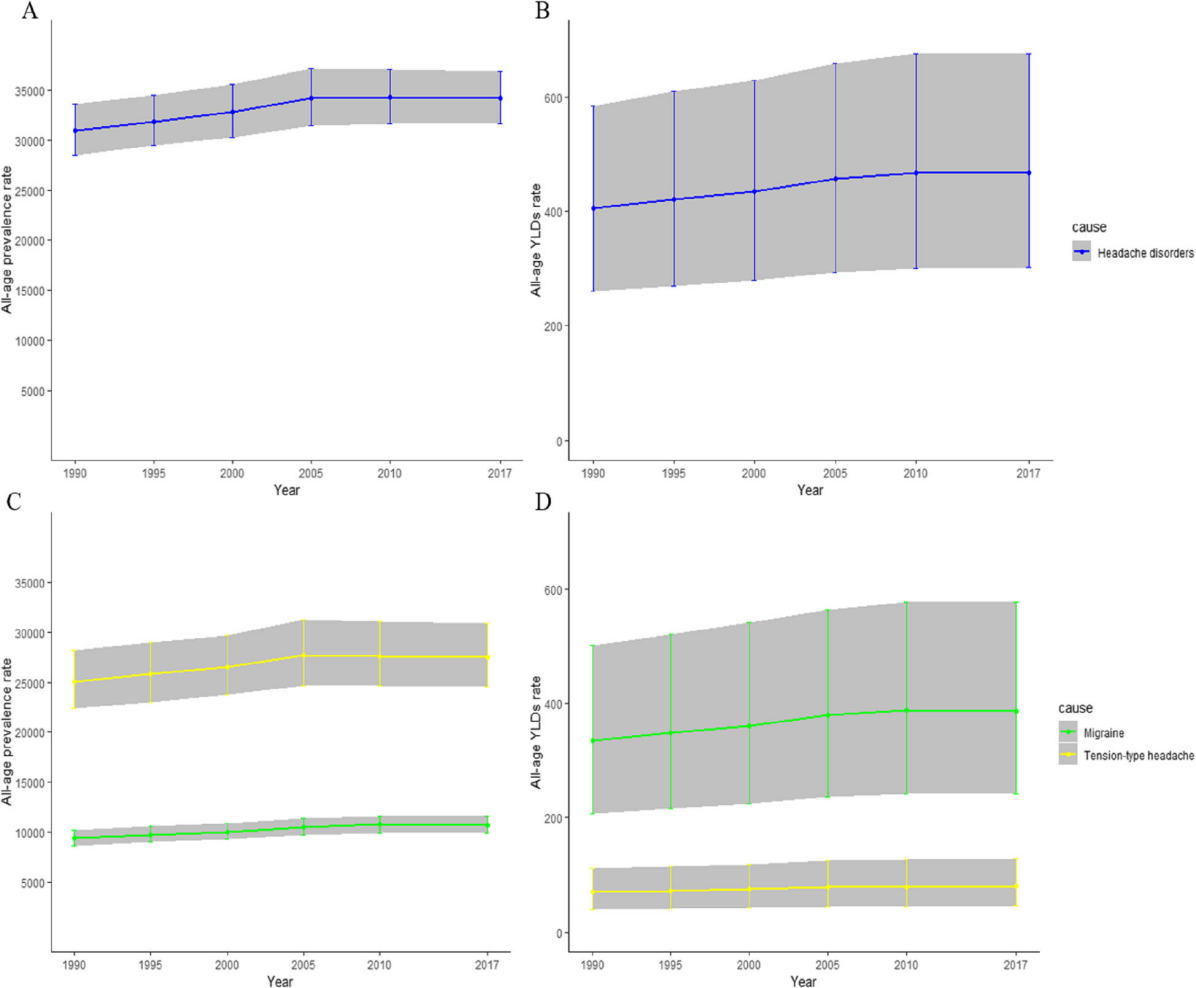
The trends of headache disorders, migraine and tension-type headache in China from 1990 to 2017. Note: **a** the all-age prevalence rate from headache disorders; **b** The all-age YLDs rate from headache disorders; **c** the all-age prevalence rate from migraine and tension-type headache; **d** The all-age YLDs rate from migraine and tension-type headache. Abbreviation: YLDs: years of life lived with disability; TTH: tension-type headache

The absolute prevalence and YLDs rate for headache disorders was 34,173 and 468 per 100,000 in 2017 in China, with 10.5% and 15.4% increase compared with 1990 respectively, as the same trend was observed for migraine and TTH. The prevalence rate of TTH was much higher than that of migraine, while the YLDs from migraine was significantly higher than that of TTH. Overall, the prevalence and YLDs rates increased steadily until 2005 and remained stable at a high level afterwards (Fig. 1).

**Fig. 2:**
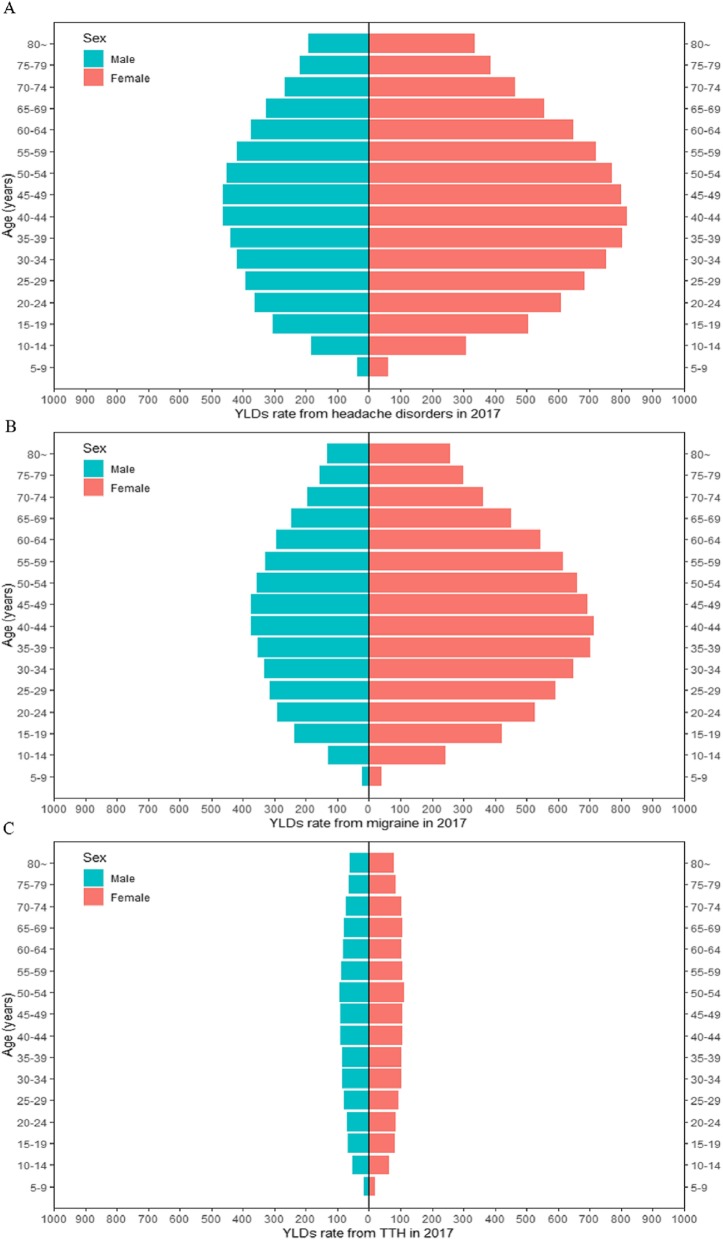
Age-sex-specific YLDs rate from headache disorders in China in 2017. Note: **a** the YLDs rate from headache disorders by age bands and sexes; **b** the YLDs rate from migraine by age bands and sexes; **c** the YLDs rate from tension-type headache by age bands and sexes. Abbreviation: YLDs: years of life lived with disability

Figure 2 showed the analysis of the age-sex-specific YLDs rate from headache disorders, migraine and TTH in China in 2017. In 2017, the YLDs rate of headache disorders, migraine, increased from younger age group and peaked at 40–44 years for both males and females. However, the YLDs rate of TTH is relatively stable from 15 to 19 years old to over 70 years old. The YLDs burden from headache disorders, migraine and TTH were higher on the population aged 30 to 54 years in 2017. For any type of headache and most age groups, the females suffered higher YLDs rate compared with males in 2017. We also showed the YLDs number by different ages and sexes in the Additional file 1: Figure S1).

**Fig. 3:**
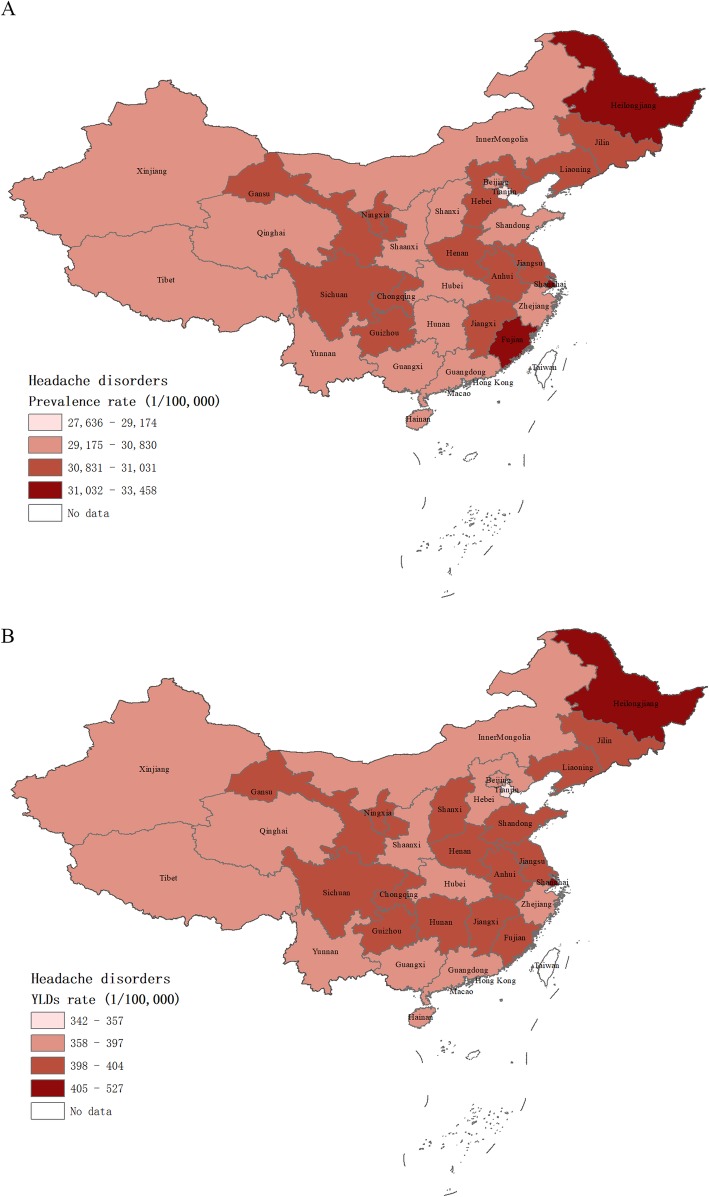
The age-standardized prevalence and YLDs rate for headache disorders in 2017 by provinces of China. Abbreviation: YLDs: years of life lived with disability. Note: **a** the age-standardized prevalence rate from headache disorders in 2017 by provinces of China; **b** the age-standardized YLDs rate from headache disorders in 2017 by provinces of China

There were disparities for age-standardized headache disorders rate across all provinces, ranging from 27,636 to 33,458 per 100,000 for prevalence, and 341 to 527 per 100,000 for YLDs in 2017 (Additional file 1: Table S1). Age-standardized prevalence and YLDs rate of migraine and TTH of each province in 2017 were shown in the Additional file 1: Table S2. As shown in Fig. 3, we observed a higher prevalence rate in eastern China than the western China. In 2017, the age-standardized prevalence rates of headache disorders were highest in Heilongjiang, Fujian and Shanghai, and age-standardized YLDs rates were highest in Heilongjiang, Shanghai and Macao SAR.

## Discussion

Few population-representative prevalence data for headache disorders were available for China. Based on the methods from GBD 2017, our findings indicate that headache disorders, in particular migraine and TTH are prevalent in China, and similar to the global level in terms of age-standardized rate of prevalence and YLDs. Although the age-standardized YLDs and prevalence rates are basically stable for both China and the globe, the substantial increase in headache cases and YLDs in Chinese population deserves more attention. After an increase of more than 30% compared with 1990, over four hundred million people had a migraine or TTH in 2017. YLDs were higher in middle-aged population aged 30~54 years and more females suffered the YLDs from headache disorders than males.

The considerable burden associated to headache disorders, migraine and TTH is connected to the huge prevalence of absolute terms, it has been increasing in the last 27 years, but when addressed in terms of age-standardized rates, it seems to be basically stable. The same happens when YLDs are taken into account: they increased in absolute terms, but are basically stable in terms of age-standardized rates. The substantial increase in headache cases and YLDs between 1990 and 2017 reflects population growth and potentially some effect of changes in the age composition (ie, fewer children, adolescents but more middle-aged people) in China. The fact that the temporal trends for age-standardized prevalence and YLDs rate remain stable may indicate that, during this period, the headache treatments have no detectable effect and few improvements. In countries with better access to health care, it would be expected to decrease on the frequency and duration of migraine attacks with treatment [2]. On the other hand, it may partly reflect how poorly the update results are available worldwide. However, in GBD, DisMod-MR 2.1 makes it possible to adjust results of studies that were done using suboptimal case definitions or methods.

A population-based door-to-door survey conducted in 2009 throughout China with 5041 respondents [4] estimated 1-year prevalence was 23.8% for primary headache disorders and 10.8% for TTH, which was lower than the results of our study in 2017 (30.9% for headache disorders and 25.2% for TTH). We believe it should be partly attributed to the relatively low sensitivity of the questionnaire for the diagnosis of headache [13], and the sample size of this study is relatively small in the Chinese population. In that door-to-door survey study, patients with migraine had worse quality of life, higher disability and higher cost compared to TTH and no-headache populations [4]. The results of our study also showed higher YLDs from migraine compared with that of TTH though the prevalence of migraine was lower than TTH. A Hungary study also showed that patients with migraine had lower quality of life compared to those with TTH [14]. With regard to the proportion of YLDs, the percent of YLDs from headache increased by 5.4% in China and 5.5% worldwide among total YLDs from all causes from 1990 to 2017 (Table 2). Thus, it can be predicted that the relative burden of headaches will further increase as the importance of other disorders, such as infections, maternal and child diseases decrease [15]. For instance, the DALYs from cardiovascular disorders and the common cancers in Chinese population such as esophageal cancer and stomach cancer decreased significantly in China [8, 16].

Prevalence rates in eastern China (the most developed and mostly urban areas) tended to be higher than the western China (the least developed and mostly rural areas), which may be due to poor lifestyle of urban residents such as stress, irregular sleep, irregular intake of meals, physical inactivity and so on [17–19]. On the other hand, there may be no clear pattern of decreasing YLDs rate of headache disorders with the socioeconomic level in China. Previous studies reported more prevalent headache in subjects with more economic problems [18], while in our study, populations of some developed coastal regions, like Fujian and Shanghai suffered the highest age-standardized prevalence rates. In general, many fatal and disabling disorders decrease with socioeconomic development, but headache disorders do not appear to be strongly associated with socioeconomic development [15]. As shown in the GBD 2017, significant reduction cannot be expected for the global burden of headache with increasing Socio-demographic Index (a measure that estimates a location’s position on a spectrum of development) [8].

For age-stratified disease burden, in general, YLDs rates for non-communicable diseases increase rapidly with age [15]. However, ageing has a less effect on overall headache disorders, migraine and TTH, as the mainly YLDs were among young and middle-aged adults, and become less prevalent with old age in China from our results. Previous study on the headache burden at global level [2] and other countries like Sweden and America [18, 20], also showed the similar results with our study. In China, previous study showed prevalence of migraine and TTH peaked during middle age (40–49 years), which were similar with our results for YLDs (peaked during 40–49 years) [4]. The American Migraine Prevalence and Prevention study is a longitudinal, population-based survey which also showed similar results. From the result of that study [20], for both females and males, the chronic migraine prevalence increased throughout adolescence, peaked in midlife, and declined after age 50 years. As for the reason of age difference in headache YLDs, perhaps headache attack is a high prevalent condition mainly during working lifetime [21]. Therefore, there is an urgent need for acceptance, education and prevent measures for middle-aged populations.

For sex-specific disease burden of headache, we observed the headache-related YLDs were higher among females than males. Previous studies showed the same results in the US [22] and Europe [23]. The review in the US confirmed those of many other studies showing that migraine and other severe or frequent headaches are far more prevalent in women than men across all racial and ethnic groups (Caucasian, Black, Hispanic and Not Hispanic) [22]. The European review on the prevalence of headache showed the headaches are more prevalent in women, and somewhat less prevalent in children and youth [23]. In previous Chinese study, headache was also less prevalent in males than in females [4]. However, the reasons for the sex difference is unclear, and more research is needed in the future to discover the reasons. We believe Chinese culture and tradition expect men to tolerate pain better, which may cause them to neglect headache, while women in China may endure higher psychological and physiological burdens.

Despite the finding in our study showed the high prevalence and high burden in China, headache disorders are under diagnosed, undertreated and under-prioritized in health-care delivery systems, and this may especially so in low- and middle-income countries. A variety of nonstandard headache diagnoses such as “vascular headache” and “nervous headache” are still widely applied in clinical practice throughout China [5]. They may lead to inappropriate treatment measures, such as analgesic drug abuse, unnecessary auxiliary examinations such as magnetic resonance imaging, and repeated consultations, which may aggravate the disease burden of headache disorders. Besides, people generally have a low self-awareness about headache presence and tend to avoid evaluation by their general practitioner, even for hospital workers [21]. Therefore, in light of the high burden of headache disorders in China for such a long time, governments and health care service providers should still pay considerable attention. The limited health care resources may be allocated to the 30 to 54 age group, which reported higher YLDs rate for headache disorders, especially for the female population. Educational programme among general practitioners, in China is now being implemented, with the aims of enhancing general practitioners’ knowledge of headache, standardizing their diagnostic and treatment approaches, as essential support for implementing headache services around the country [24].

Major limitations still exist in the GBD 2017 about headache disorders in China. The most notable one is the short supply of epidemiological data from China, the paucity of studies giving data on average time which headache attack, and great methodological heterogeneity. Although the GBD collaborations made great effort to collect all published and unpublished data, the quantity and quality of data available about headache are still limited, which could affect the accuracy of the estimated burden. However, the paucity of data and methodology heterogeneity in estimating headache prevalence can be evaluated and addressed accordingly by DisMod 2.1 which was developed for the GBD 2017 study and the validation had been previously published [8]. In addition, we only focus on the disparities of burden from headache by age, sex and regions, while ignoring other factors, such as school-goers versus non-school-goers, and urban versus rural areas. Some studies have shown headache is common among school-goers and associated with lower quality of life and poor academic performance [25, 26]. Therefore, we need further work to provide more detailed information of burden of headache.

## Conclusion

In summary, this study demonstrates a huge health burden from headache disorders, and progress in controlling headache burden in China has been suboptimal over the last 27 years. Effective policies and measures to address this rising burden should be a national priority to further improve the quality of Chinese citizens, especially for female and middle-aged adults.

## Supplementary information

**Additional file 1. **Method. Further details on the methods of prevalence and YLDs estimates. **Figure S1.** Age-sex-specific YLDs number from headache disorders in China. **Table S1.** Age-standardized prevalence rate and YLDs rate from 1990 to 2017 for total headache disorders by province of China. **Table S2.** Age-standardized prevalence rate and YLDs rate from 1990 to 2017 for specific headache disorders by province of China.

## Data Availability

All related data can searched and extracted from the website database (http://ghdx.healthdata.org), which was set up and shared by the GBD group.

## Abbreviations

DALYs: Disability-adjusted life years
DisMod-MR 2.1: Disease Modeling-Meta regression 2.1
GBD: The Global Burden of Diseases, Injuries, and Risk Factors study
ICHD: International Classification of Headache Diseases
SAR: Special Administrative Regions
TTH: Tension-type headache
UI: The uncertainty interval
YLDs: Years of life lived with disability
