# Supplementary material for: Burden of headache disorders in China, 1990–2017: findings from the Global Burden of Disease Study 2017
Source: J Headache Pain. 2019 Nov 7;20(1):102. doi: 10.1186/s10194-019-1048-2 (PMC6836347; doi:10.1186/s10194-019-1048-2)
Supplement: Supplementary file 1 — Additional file 1. Method. Further details on the methods of prevalence and YLDs estimates. Figure S1. Age-sex-specific YLDs number from headache disorders in China. Table S1. Age-standardized prevalence rate and YLDs rate from 1990 to 2017 for total headache disorders by province of China. Table S2. Age-standardized prevalence rate and YLDs rate from 1990 to 2017 for specific headache disorders by province of China. [file 10194_2019_1048_MOESM1_ESM.docx]

**Appendix:**

**Method:** Further details on the methods of prevalence and YLDs estimates.

**Figure S1:** Age-sex-specific YLDs number from headache disorders in China.

**Table S1:** Age-standardized prevalence rate and YLDs rate from 1990 to 2017 for total headache disorders by province of China.

**Table S2:** Age-standardized prevalence rate and YLDs rate from 1990 to 2017 for specific headache disorders by province of China.

**Method:** **Further details on the methods of prevalence and YLDs estimates**.

All analyses were based on China data in the GBD study 2017. Details on the data, approaches to enhancing data quality and comparability, and statistical modelling and metrics for the GBD 2017 is published elsewhere [1, 2]. Further details on the methods of prevalence and YLDs estimates were presented as followed.

**1. Data sources**

The data sources for headache disorders were mainly based on published population-based studies of prevalence and survey data for which we had the individual record data were also included [1, 3]. In addition, hospital claims data could provide more detailed information on age patterns, time trends and spatial patterns on subnational locations than published epidemiological studies, therefore are also included. For the previous population-based studies, we only included the study of headache diagnosis which based on the International Classification of Headache Diseases (ICHD). The ICHD was first published in 1988, and after the second edition of ICHD [4] updated in 2004, the third edition of the International Classification of Headache Disorders beta (ICHD-3 beta) has now been published in 2013 [5]. They have been widely accepted and should now form the basis of all diagnosis and management of headache disorders in clinical practice as well as in research [6]. The ICHD-3 beta, like its predecessors, is hierarchical and allows diagnosis in different clinical settings. In China, the diagnosis of headache disorders based on ICHD-3 beta has been proved with high diagnostic accuracy for most of the primary and some types of secondary headaches [7]. We only included the headache type of medication overuse headache, migraine and TTH, then the reference lists in published articles were reviewed and data were solicited from GBD network of more than 2500 collaborators. As for some other secondary headaches, like infections or brain tumors related headache, the diagnosis is difficult in epidemiological studies, and the burden of these headaches should be attributed to the underlying disorder [8].

**2. Prevalence modelling and correct step**

The detailed descriptions of the modeling strategy for point prevalence and YLDs estimation and validation had been previously published [1]. Prevalence data were matched by headache type, age, sex, year and location. Disease Modeling-Meta regression 2.1 (DisMod-MR 2.1), was developed for the GBD 2017 study to address the challenges in estimating the point prevalence and YLDs outcomes in all regions and countries, and estimates were obtained in this way also for regions where no relevant headache studies had been done. Among the data sources used in GBD 2017, the prevalence rates vary across the world, but the degree to which this reflects real variation across borders and time, or method, is mostly not known. We consider the methodological differences may play a significant role because the results may be substantially affected by relatively minor differences, such as diversities in screening question [9]. To adjust for differences in methodological quality, all included epidemiological studies are scored according to the revised edition (dichotomy variables) of the published methodological quality standards for headache epidemiological studies [8]. In DisMod-MR 2.1, these methodological variables were evaluated for a systematic difference and corrected accordingly.

**3. Calculation of prevalence and YLDs**

The prevalence reflects the individuals in the population who have had at least one episode in the past 12 months fulfilling ICHD-3 beta criteria. The data on frequency and duration of headache attacks were identified from population-based studies. From these data, we estimated the average number of hours patients spend in headache attacks, and expressed this as a proportion of a year [3]. YLDs are estimated as the product of prevalence and the mean time patients spend with that type of headache (or sequelae) multiplied by their corresponding disability weights through population and internet surveys [3], which quantify the relative severity of sequelae as a number between 0 (representing full health) and 1 (representing death) [10].

**References:**

1. GBD 2017 Disease and Injury Incidence and Prevalence Collaborators (2018) Global, regional, and national incidence, prevalence, and years lived with disability for 354 diseases and injuries for 195 countries and territories, 1990-2017: a systematic analysis for the Global Burden of Disease Study 2017. Lancet 392(10159):1789-858.

2. GBD 2017 Causes of Death Collaborators (2018) Global, regional, and national age-sex-specific mortality for 282 causes of death in 195 countries and territories, 1980-2017: a systematic analysis for the Global Burden of Disease Study 2017. Lancet 392(10159):1736-88.

3. GBD 2016 Headache Collaborators (2018) Global, regional, and national burden of migraine and tension-type headache, 1990-2016: a systematic analysis for the Global Burden of Disease Study 2016. Lancet Neurol 17(11):954-76.

4. Headache Classification Committee of the International Headache Society (IHS) (2004) The International Classification of Headache Disorders: 2nd edition. Cephalalgia 24 Suppl 1:9-160.

5. Headache Classification Committee of the International Headache Society (IHS) (2013) The International Classification of Headache Disorders, 3rd edition (beta version). Cephalalgia 33(9):629-808.

6. Headache Classification Committee of the International Headache Society (IHS) (2018) Headache Classification Committee of the International Headache Society (IHS) The International Classification of Headache Disorders, 3rd edition. Cephalalgia 38(1):1-211.

7. Dong Z, Yin Z, He M, Chen X, Lv X, Yu S (2014) Validation of a guideline-based decision support system for the diagnosis of primary headache disorders based on ICHD-3 beta. J Headache Pain 15:40.

8. Stovner LJ, Al JM, Birbeck GL, Gururaj G, Jensen R, Katsarava Z *et al* (2014) The methodology of population surveys of headache prevalence, burden and cost: principles and recommendations from the Global Campaign against Headache. J Headache Pain 15:5.

9. Hagen K, Zwart JA, Aamodt AH, Nilsen KB, Brathen G, Helde G *et al* (2008) A face-to-face interview of participants in HUNT 3: the impact of the screening question on headache prevalence. J Headache Pain 9(5):289-94.

10. GBD 2016 Neurological Disorders Collaborator Group (2019) Global, regional, and national burden of neurological disorders, 1990-2016: a systematic analysis for the Global Burden of Disease Study 2016. Lancet Neurol 18(5):459-80.


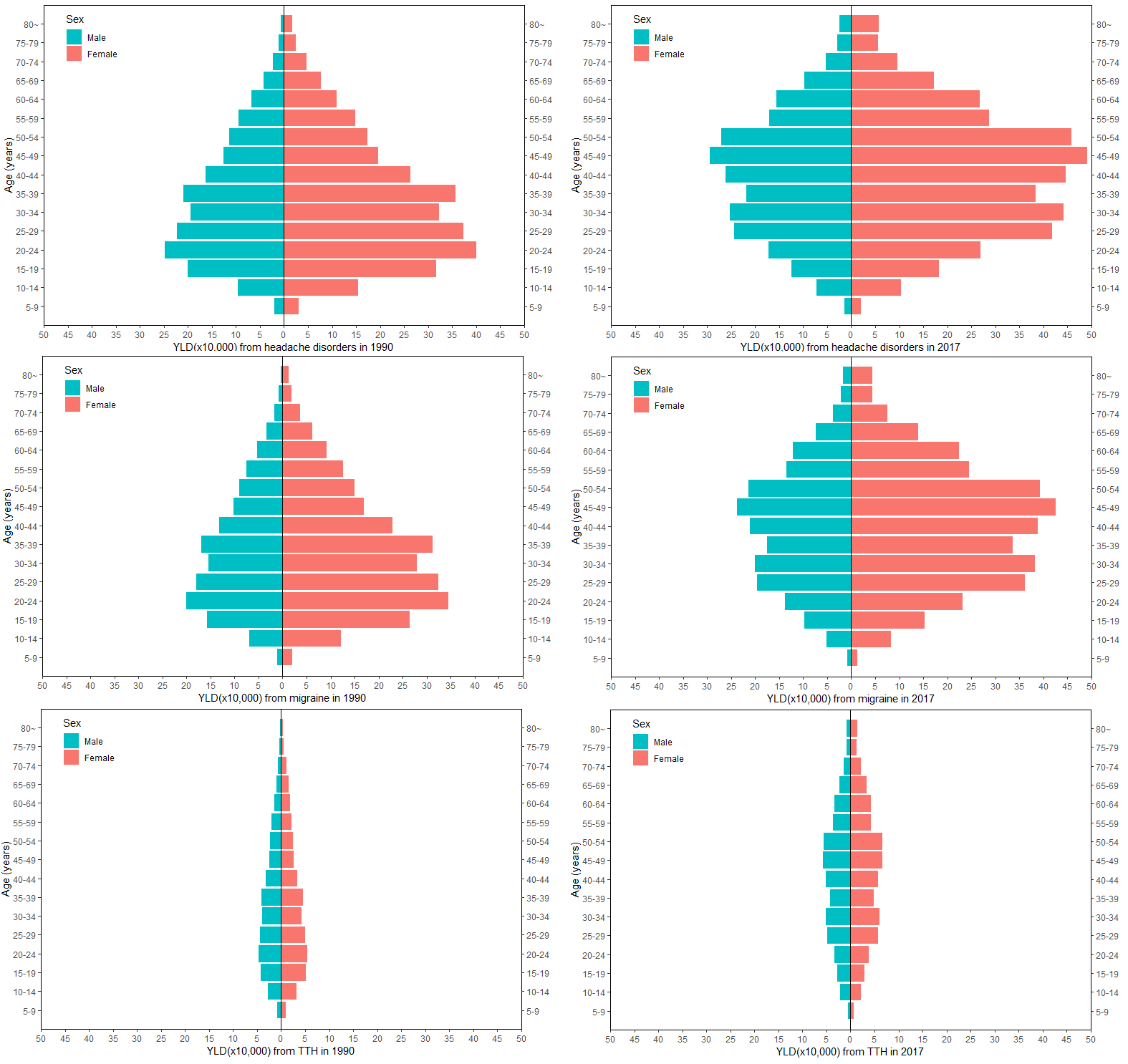
**Figure S1:** Age-sex-specific YLDs number from headache disorders in China.

| Table S1. Age-standardized prevalence rate and YLDs rate from 1990 to 2017 for total headache disorders by province of China | | | | |
| --- | --- | --- | --- | --- |
| Region | YLDs rate per 100,000 (95% UI) | | Prevalence rate per 100,000 (95% UI) | |
|  | 1990 | 2017 | 1990 | 2017 |
| China | 402.0(258.9 - 580.8) | 401.2(259.1 - 577.3) | 30,860.4(28,665.5 - 33,381.4) | 30,936.3(28,626.6 - 33,430.5) |
| Provinces |  |  |  |  |
| Anhui | 396.3(254.3 - 573.5) | 399.0(257.8 - 576.5) | 30,766.1(28,536.6 - 33,298.3) | 30,884.8(28,546.5 - 33,443.3) |
| Beijing | 398.6(256.5 - 570.9) | 395.0(254.4 - 568.8) | 30,738.6(28,565.0 - 33,243.9) | 30,804.0(28,496.6 - 33,326.8) |
| Chongqing | 395.5(257.2 - 570.0) | 401.2(259.9 - 581.0) | 30,698.4(28,503.5 - 33,214.6) | 30,939.7(28,605.5 - 33,511.4) |
| Fujian | 400.7(257.0 - 583.3) | 400.8(259.5 - 576.9) | 32,162.3(29,925.0 - 34,837.6) | **32,468.1(30,079.5 - 35,151.8)** |
| Gansu | 395.7(258.1 - 572.4) | 398.2(257.8 - 575.9) | 30,705.1(28,494.2 - 33,261.7) | 30,868.9(28,531.2 - 33,429.5) |
| Guangdong | 398.4(257.6 - 574.8) | 392.5(255.2 - 566.6) | 30,814.7(28,600.1 - 33,358.3) | 30,676.9(28,342.0 - 33,205.4) |
| Guangxi | 396.0(254.2 - 568.6) | 396.5(255.7 - 575.7) | 30,693.5(28,477.3 - 33,248.9) | 30,807.5(28,469.6 - 33,304.4) |
| Guizhou | 394.7(255.4 - 568.3) | 397.6(258.8 - 575.8) | 30,718.9(28,542.9 - 33,237.0) | 30,886.2(28,544.3 - 33,439.0) |
| Hainan | 396.4(257.1 - 577.8) | 393.6(254.7 - 566.9) | 30,762.7(28,570.2 - 33,252.1) | 30,675.4(28,387.6 - 33,283.8) |
| Hebei | 399.5(258.4 - 580.2) | 396.1(257.6 - 569.9) | 30,791.6(28,630.3 - 33,305.0) | 30,854.6(28,524.0 - 33,403.2) |
| Heilongjiang | 526.5(341.2 - 764.9) | **527.0(339.2 - 758.4)** | 33,286.8(31,211.3 - 35,748.5) | **33,457.9(31,264.4 - 35,868.5)** |
| Henan | 399.5(258.3 - 577.7) | 401.0(258.1 - 577.2) | 30,810.4(28,604.6 - 33,359.7) | 30,933.4(28,644.7 - 33,484.7) |
| Hubei | 396.0(254.7 - 571.8) | 396.1(254.9 - 568.0) | 30,755.1(28,552.2 - 33,297.3) | 30,810.9(28,495.3 - 33,348.0) |
| Hunan | 395.7(257.1 - 570.6) | 398.1(257.7 - 575.8) | 30,672.2(28,467.3 - 33,214.2) | 30,825.5(28,521.8 - 33,338.4) |
| Inner Mongolia | 396.2(255.9 - 575.8) | 394.8(254.6 - 568.5) | 30,611.7(28,433.4 - 33,109.6) | 30,745.6(28,418.0 - 33,262.8) |
| Jiangsu | 398.9(258.3 - 575.4) | 397.8(258.3 - 576.7) | 30,820.8(28,609.5 - 33,311.5) | 30,842.8(28,492.8 - 33,376.4) |
| Jiangxi | 396.6(255.7 - 575.3) | 398.5(259.0 - 574.4) | 30,741.1(28,546.6 - 33,235.0) | 30,837.3(28,505.2 - 33,273.3) |
| Jilin | 399.7(258.8 - 572.6) | 399.3(259.2 - 578.5) | 30,720.4(28,515.4 - 33,237.1) | 30,867.6(28,548.1 - 33,368.5) |
| Liaoning | 399.4(259.8 - 577.5) | 397.6(257.6 - 573.0) | 30,741.9(28,539.8 - 33,291.2) | 30,840.1(28,508.4 - 33,407.6) |
| Ningxia | 398.2(257.5 - 576.9) | 398.0(256.3 - 575.6) | 30,685.9(28,471.3 - 33,207.3) | 30,841.8(28,510.2 - 33,398.8) |
| Qinghai | 397.6(259.8 - 580.3) | 395.7(257.5 - 571.7) | 30,723.0(28,539.1 - 33,208.2) | 30,780.6(28,477.4 - 33,297.9) |
| Shaanxi | 398.4(258.1 - 574.8) | 395.9(256.5 - 571.7) | 30,694.6(28,473.3 - 33,210.8) | 30,776.7(28,463.2 - 33,295.9) |
| Shandong | 400.6(261.2 - 575.6) | 397.5(257.7 - 575.8) | 30,830.5(28,611.2 - 33,353.8) | 30,829.6(28,497.0 - 33,331.4) |
| Shanghai | 457.9(290.9 - 686.3) | **455.2(292.6 - 677.6)** | 32,235.4(30,040.0 - 34,753.2) | **32,204.2(29,971.0 - 34,646.2)** |
| Shanxi | 396.2(257.7 - 575.7) | 397.3(256.5 - 574.7) | 30,684.9(28,488.2 - 33,224.5) | 30,827.5(28,457.1 - 33,314.6) |
| Sichuan | 396.3(257.8 - 573.6) | 397.7(258.3 - 574.3) | 30,721.2(28,527.0 - 33,265.7) | 30,894.0(28,566.9 - 33,429.5) |
| Tianjin | 367.8(239.0 - 534.5) | 356.7(234.2 - 513.7) | 29,484.7(26,431.5 - 32,705.3) | 29,173.9(26,142.9 - 32,518.1) |
| Tibet | 400.3(258.1 - 574.7) | 396.1(256.8 - 573.3) | 30,924.2(28,722.4 - 33,423.6) | 30,820.7(28,438.8 - 33,351.5) |
| Xinjiang | 397.6(257.2 - 573.0) | 396.3(257.4 - 573.5) | 30,619.6(28,445.2 - 33,150.1) | 30,783.4(28,481.0 - 33,339.5) |
| Yunnan | 397.4(258.7 - 578.1) | 395.9(254.8 - 573.6) | 30,775.7(28,574.5 - 33,350.0) | 30,803.4(28,498.8 - 33,323.4) |
| Zhejiang | 396.0(257.2 - 564.8) | 396.0(255.4 - 571.1) | 30,724.2(28,547.4 - 33,262.9) | 30,777.6(28,428.0 - 33,340.3) |
| Special Administrative Region | | |  |  |
| Hong Kong | 331.1(215.8 - 484.5) | 341.7(223.2 - 504.1) | 26,878.7(24,416.5 - 29,440.4) | 27,636.2(25,092.8 - 30,380.0) |
| Macao | 405.8(263.5 - 586.5) | **404.1(262.9 - 581.2)** | 31,005.3(28,801.3 - 33,511.2) | 31,031.5(28,712.1 - 33,601.0) |
| Note: YLDs = years lived with disability | | | | |
| Age-standardized rate (1/100,000); Data shown as rate (95% uncertainty interval); The highest rate were highlighted in bold. | | | | |

| Table S2. Age-standardized prevalence rate and YLDs rate from 1990 to 2017 for specific headache disorders by province of China | | | | | |
| --- | --- | --- | --- | --- | --- |
| Region |  | YLDs rate per 100,000 (95% UI) | | Prevalence rate per 100,000 (95% UI) | |
|  |  | 1990 | 2017 | 1990 | 2017 |
| China | Migraine | 332.0(206.3 - 495.0) | 331.1(207.0 - 494.6) | 9267.5(8601.9 - 10007.7) | 9211.2(8527.0 - 9921.4) |
|  | TTH | 70.0(39.5 - 112.5) | 70.1(39.4 - 113.3) | 25098.8(22595.0 - 28046.4) | 25232.7(22610.8 - 28147.9) |
| Provinces |  |  |  |  |  |
| Anhui | Migraine | 326.4(203.8 - 489.4) | 328.8(205.5 - 491.2) | 9111.0(8440.8 - 9838.7) | 9138.5(8456.4 - 9842.1) |
|  | TTH | 69.9(38.8 - 111.6) | 70.1(39.1 - 111.9) | 25097.9(22625.3 - 28038.5) | 25225.9(22605.2 - 28116.2) |
| Beijing | Migraine | 328.5(204.1 - 491.0) | 325.2(202.5 - 487.1) | 9137.9(8471.3 - 9866.9) | 9044.1(8368.1 - 9736.3) |
|  | TTH | 70.0(39.1 - 112.6) | 69.8(39.1 - 112.0) | 25054.6(22567.9 - 27977.2) | 25197.9(22593.2 - 28082.1) |
| Chongqing | Migraine | 325.7(202.4 - 484.7) | 330.9(206.5 - 492.7) | 9083.7(8417.7 - 9809.5) | 9177.7(8493.3 - 9887.4) |
|  | TTH | 69.9(38.9 - 112.0) | 70.3(39.0 - 114.0) | 25047.4(22560.5 - 27982.1) | 25262.5(22636.9 - 28154.9) |
| Fujian | Migraine | 327.9(203.8 - 491.9) | 327.4(203.2 - 490.6) | 9120.9(8449.7 - 9850.9) | 9049.8(8377.7 - 9742.1) |
|  | TTH | 72.9(40.4 - 118.1) | 73.4(40.6 - 120.0) | 26776.4(24143.2 - 29837.1) | 27212.2(24492.7 - 30360.1) |
| Gansu | Migraine | 325.9(203.6 - 488.1) | 328.1(206.5 - 489.3) | 9107.7(8440.9 - 9837.7) | 9116.1(8435.8 - 9817.1) |
|  | TTH | 69.7(38.9 - 110.6) | 70.0(38.9 - 112.5) | 25037.2(22548.1 - 27969.5) | 25225.8(22607.6 - 28122.4) |
| Guangdong | Migraine | 328.3(203.6 - 492.8) | 322.8(203.3 - 484.9) | 9148.5(8477.2 - 9878.2) | 8958.3(8297.4 - 9644.5) |
|  | TTH | 70.1(39.6 - 111.3) | 69.7(38.5 - 113.6) | 25125.9(22651.5 - 28066.6) | 25116.8(22516.6 - 27993.5) |
| Guangxi | Migraine | 326.1(201.2 - 485.4) | 326.5(204.7 - 493.5) | 9079.4(8415.1 - 9802.7) | 9050.0(8375.6 - 9742.8) |
|  | TTH | 69.9(38.6 - 112.1) | 70.1(39.1 - 112.8) | 25042.7(22555.2 - 27968.6) | 25198.2(22592.8 - 28089.2) |
| Guizhou | Migraine | 325.1(203.0 - 484.3) | 327.6(204.8 - 490.0) | 9110.9(8444.1 - 9839.2) | 9114.6(8431.9 - 9819.6) |
|  | TTH | 69.6(38.9 - 111.1) | 70.0(39.0 - 113.0) | 25051.7(22564.2 - 27982.7) | 25239.9(22628.6 - 28140.5) |
| Hainan | Migraine | 326.5(203.4 - 489.4) | 323.7(203.9 - 487.1) | 9105.3(8440.4 - 9828.8) | 8956.2(8288.7 - 9647.6) |
|  | TTH | 69.9(39.0 - 112.3) | 69.8(38.9 - 111.8) | 25099.5(22628.5 - 28031.3) | 25116.1(22517.9 - 27985.7) |
| Hebei | Migraine | 329.3(204.4 - 494.5) | 326.2(202.8 - 487.8) | 9159.8(8487.6 - 9893.2) | 9093.5(8417.2 - 9789.5) |
|  | TTH | 70.2(38.8 - 112.2) | 69.9(39.0 - 112.8) | 25091.2(22607.7 - 28031.4) | 25218.5(22605.7 - 28117.7) |
| Heilongjiang | Migraine | 456.7(286.5 - 674.7) | 456.9(285.7 - 675.9) | 13207.2(12238.1 - 14240.5) | 13194.5(12257.2 - 14195.0) |
|  | TTH | 69.8(38.9 - 111.3) | 70.0(38.8 - 112.1) | 25012.4(22516.2 - 27948.5) | 25223.8(22609.8 - 28123.5) |
| Henan | Migraine | 329.3(204.8 - 491.2) | 330.7(206.8 - 495.0) | 9158.0(8485.4 - 9889.6) | 9176.0(8494.9 - 9885.1) |
|  | TTH | 70.2(39.2 - 111.7) | 70.3(39.5 - 113.4) | 25117.9(22642.1 - 28056.3) | 25256.1(22635.8 - 28142.3) |
| Hubei | Migraine | 326.1(202.8 - 487.9) | 326.3(204.1 - 487.2) | 9119.8(8451.0 - 9849.2) | 9076.1(8402.9 - 9772.4) |
|  | TTH | 69.9(38.9 - 111.4) | 69.8(39.0 - 111.8) | 25081.0(22598.5 - 28015.5) | 25189.0(22576.5 - 28079.1) |
| Hunan | Migraine | 325.9(203.8 - 485.0) | 328.0(206.1 - 491.7) | 9075.0(8408.8 - 9802.7) | 9081.1(8403.8 - 9776.9) |
|  | TTH | 69.8(38.5 - 112.1) | 70.0(39.2 - 113.1) | 25021.6(22527.8 - 27954.3) | 25196.0(22582.5 - 28092.2) |
| Inner Mongolia | Migraine | 326.5(201.7 - 489.5) | 324.8(201.8 - 484.0) | 9075.4(8411.0 - 9809.4) | 8994.8(8324.9 - 9684.6) |
|  | TTH | 69.8(39.2 - 112.4) | 69.9(39.1 - 112.7) | 24956.0(22467.2 - 27887.3) | 25163.9(22559.5 - 28053.2) |
| Jiangsu | Migraine | 328.8(204.0 - 495.2) | 327.8(203.2 - 492.0) | 9169.5(8497.0 - 9899.8) | 9091.6(8419.1 - 9788.6) |
|  | TTH | 70.1(38.5 - 112.1) | 70.0(39.1 - 112.4) | 25128.1(22652.3 - 28065.5) | 25207.5(22594.2 - 28103.7) |
| Jiangxi | Migraine | 326.7(201.8 - 487.8) | 328.3(205.4 - 489.6) | 9112.7(8444.0 - 9841.5) | 9099.0(8422.6 - 9798.0) |
|  | TTH | 69.9(39.3 - 111.3) | 70.1(38.9 - 113.9) | 25071.6(22589.6 - 28005.3) | 25200.9(22588.4 - 28089.9) |
| Jilin | Migraine | 329.6(205.8 - 490.3) | 329.2(204.8 - 490.0) | 9152.2(8482.8 - 9888.6) | 9104.0(8424.9 - 9802.9) |
|  | TTH | 70.1(39.6 - 111.9) | 70.2(39.2 - 113.6) | 25029.9(22532.7 - 27964.9) | 25231.7(22617.2 - 28131.9) |
| Liaoning | Migraine | 329.4(205.6 - 492.8) | 327.5(206.6 - 495.1) | 9159.6(8489.5 - 9894.8) | 9079.9(8404.7 - 9775.3) |
|  | TTH | 70.0(39.0 - 111.7) | 70.1(38.7 - 113.3) | 25046.5(22552.6 - 27982.2) | 25213.1(22599.5 - 28112.3) |
| Ningxia | Migraine | 328.3(203.2 - 489.6) | 327.8(205.5 - 496.2) | 9124.5(8457.9 - 9859.8) | 9078.1(8400.1 - 9777.0) |
|  | TTH | 69.9(39.3 - 111.4) | 70.2(39.2 - 112.3) | 25007.2(22513.1 - 27942.4) | 25215.5(22603.2 - 28114.2) |
| Qinghai | Migraine | 327.6(204.6 - 491.7) | 325.7(204.7 - 488.6) | 9105.9(8441.4 - 9833.0) | 9015.9(8342.6 - 9709.3) |
|  | TTH | 70.0(39.1 - 111.9) | 70.0(38.8 - 111.9) | 25055.2(22558.1 - 27982.4) | 25188.2(22585.3 - 28074.3) |
| Shaanxi | Migraine | 328.4(203.5 - 491.2) | 325.9(204.2 - 488.3) | 9113.1(8446.3 - 9844.5) | 9037.2(8365.6 - 9728.5) |
|  | TTH | 70.0(39.4 - 112.2) | 70.1(39.2 - 112.7) | 25018.8(22528.6 - 27950.9) | 25175.5(22568.2 - 28062.6) |
| Shandong | Migraine | 330.3(206.3 - 493.4) | 327.4(205.0 - 490.6) | 9183.5(8510.0 - 9916.6) | 9072.1(8400.0 - 9764.9) |
|  | TTH | 70.3(39.5 - 112.6) | 70.1(39.3 - 112.9) | 25121.3(22640.1 - 28062.6) | 25207.3(22597.5 - 28105.2) |
| Shanghai | Migraine | 387.9(236.0 - 603.2) | 385.5(234.8 - 592.4) | 11362.3(10567.3 - 12235.5) | 11245.3(10434.4 - 12079.3) |
|  | TTH | 70.0(38.9 - 112.9) | 69.8(38.9 - 113.0) | 25096.8(22609.6 - 28019.1) | 25155.4(22549.4 - 28042.2) |
| Shanxi | Migraine | 326.3(203.5 - 486.6) | 327.3(203.2 - 489.2) | 9091.0(8424.6 - 9819.4) | 9067.3(8390.8 - 9765.4) |
|  | TTH | 69.9(39.0 - 111.7) | 70.0(38.7 - 112.9) | 25027.3(22533.9 - 27959.9) | 25204.2(22591.4 - 28103.9) |
| Sichuan | Migraine | 326.4(204.5 - 487.7) | 327.7(204.0 - 489.1) | 9098.4(8432.2 - 9825.1) | 9126.4(8445.8 - 9828.8) |
|  | TTH | 69.9(39.2 - 112.0) | 70.0(39.4 - 112.1) | 25059.7(22574.4 - 27995.0) | 25239.3(22621.8 - 28137.1) |
| Tianjin | Migraine | 298.7(186.9 - 450.0) | 288.3(180.9 - 434.5) | 8114.3(7068.4 - 9272.7) | 7794.7(6842.2 - 8907.3) |
|  | TTH | 69.1(37.9 - 109.5) | 68.4(37.6 - 109.4) | 24386.0(21048.7 - 27870.1) | 24267.6(20884.9 - 28044.1) |
| Tibet | Migraine | 330.1(207.7 - 491.1) | 326.0(203.9 - 488.0) | 9238.5(8564.8 - 9970.6) | 9038.5(8367.2 - 9727.2) |
|  | TTH | 70.1(38.8 - 112.5) | 70.0(39.5 - 112.6) | 25186.7(22702.4 - 28121.5) | 25216.8(22615.2 - 28104.5) |
| Xinjiang | Migraine | 327.7(203.6 - 487.7) | 326.3(202.7 - 487.0) | 9096.8(8430.6 - 9834.9) | 25179.7(22563.9 - 28076.1) |
|  | TTH | 69.9(39.1 - 111.2) | 70.0(39.4 - 113.0) | 24949.6(22463.9 - 27884.7) | 9037.9(8362.3 - 9737.5) |
| Yunnan | Migraine | 327.4(204.4 - 490.8) | 325.9(204.6 - 491.9) | 9141.5(8472.5 - 9871.2) | 9044.2(8370.2 - 9736.1) |
|  | TTH | 70.0(39.1 - 111.9) | 70.0(39.2 - 113.5) | 25090.2(22604.2 - 28022.5) | 25194.4(22586.6 - 28087.6) |
| Zhejiang | Migraine | 326.2(203.1 - 487.1) | 326.0(203.2 - 485.4) | 9115.8(8445.6 - 9848.4) | 9032.8(8361.9 - 9724.0) |
|  | TTH | 69.8(38.8 - 110.9) | 69.9(39.1 - 112.6) | 25053.4(22569.2 - 27993.7) | 25174.7(22564.0 - 28068.9) |
| Special Administrative Region | | |  |  |  |
| Hong Kong | Migraine | 265.4(165.2 - 405.4) | 275.2(171.7 - 425.4) | 7109.9(6326.4 - 7975.7) | 7422.4(6569.5 - 8326.2) |
|  | TTH | 65.7(36.7 - 104.2) | 66.6(36.8 - 106.1) | 22204.2(19505.8 - 25003.2) | 22833.2(19973.3 - 25953.0) |
| Macao | Migraine | 335.0(208.6 - 504.0) | 333.6(208.8 - 501.3) | 9279.2(8593.9 - 10015.3) | 9237.2(8554.9 - 9952.4) |
|  | TTH | 70.8(39.7 - 113.5) | 70.5(39.3 - 113.9) | 25243.0(22778.6 - 28201.1) | 25315.9(22683.9 - 28214.2) |
| Note: YLDs = years lived with disability | | | | | |
| Age-standardized rate (1/100,000) and data shown as rate (95% uncertainty interval) | | | | | |
